# Plant-Based Production of Recombinant Plasmodium Surface Protein Pf38 and Evaluation of its Potential as a Vaccine Candidate

**DOI:** 10.1371/journal.pone.0079920

**Published:** 2013-11-21

**Authors:** Tatjana Feller, Pascal Thom, Natalie Koch, Holger Spiegel, Otchere Addai-Mensah, Rainer Fischer, Andreas Reimann, Gabriele Pradel, Rolf Fendel, Stefan Schillberg, Matthias Scheuermayer, Helga Schinkel

**Affiliations:** 1 Fraunhofer Institute for Molecular Biology and Applied Ecology (IME), Aachen, Germany; 2 RWTH Aachen University, Institute for Molecular Biotechnology, Aachen, Germany; 3 Research Center for Infectious Diseases, University of Wuerzburg, Wuerzburg, Germany; Pasteur Institute Lille, France

## Abstract

Pf38 is a surface protein of the malarial parasite *Plasmodium falciparum*. In this study, we produced and purified recombinant Pf38 and a fusion protein composed of red fluorescent protein and Pf38 (RFP-Pf38) using a transient expression system in the plant *Nicotiana benthamiana*. To our knowledge, this is the first description of the production of recombinant Pf38. To verify the quality of the recombinant Pf38, plasma from semi-immune African donors was used to confirm specific binding to Pf38. ELISA measurements revealed that immune responses to Pf38 in this African subset were comparable to reactivities to AMA-1 and MSP1_19_. Pf38 and RFP-Pf38 were successfully used to immunise mice, although titres from these mice were low (on average 1∶11.000 and 1∶39.000, respectively). In immune fluorescence assays, the purified IgG fraction from the sera of immunised mice recognised Pf38 on the surface of schizonts, gametocytes, macrogametes and zygotes, but not sporozoites. Growth inhibition assays using αPf38 antibodies demonstrated strong inhibition (≥60%) of the growth of blood-stage *P. falciparum*. The development of zygotes was also effectively inhibited by αPf38 antibodies, as determined by the zygote development assay. Collectively, these results suggest that Pf38 is an interesting candidate for the development of a malaria vaccine.

## Introduction

Malaria continues to be a major health burden worldwide. In 2010, 216 million estimated cases and 655.000 deaths from malaria were reported. Ninety-one percent of these malaria cases were due to infection with *P. falciparum* [1]. Although increasing knowledge about the parasite and its interaction with its two host organisms, humans and mosquitoes, has amassed, a viable strategy to prevent the disease has yet to be identified. Current efforts to control malaria, including insecticide-treated mosquito nets, spraying of long-lasting insecticides [2] and artemisinin-based combination therapy as a first treatment [3], diminish malaria incidence but continue to leave a high burden of this disease in sub-Saharan areas [4]. Additionally, imperfect diagnostics and self-medication [5] have led to inaccurate data collection and an underestimation of malaria cases. Furthermore, resistance among malaria vectors (e.g., resistance to pyrethroid insecticides) has been detected in many countries in sub-Saharan Africa [6], indicating that moderately effective measures today could become useless in the future.

A desirable means to control malaria would be a vaccine capable of eliciting a protective immune response, and numerous approaches to develop such a malaria vaccine have been pursued over the last 70 years [7], [8]. It is assumed essential that such a vaccine would not only prevent clinical symptoms but also inhibit the transmission of the disease-causing parasites via mosquitoes to completely eradicate the disease [9], [10]. No commercially available malaria vaccines exist to date. Although there are candidate vaccines in clinical trials, it is unclear whether these will be sufficiently effective at preventing disease, particularly in regard to mid- and long-term efficacy [11]–[13].

In the process of searching for a candidate for a new malaria vaccine, we became interested in Pf38 (GenBank accession ABO41470.1), which was proposed as a potential vaccine candidate in 2005 [14]. Pf38 is a glycosylphosphatidylinositol (GPI)-anchored merozoite surface protein [14] that accounts for 5% of the total GPI-anchored proteins on the merozoite surface and is concentrated in apical secretion organelles in early schizonts [15]. Pf38 belongs to the six-cysteine family (6-cys), a conserved, apicomplexan specific protein family characterized by cystein rich motives sharing a common spacing pattern of the 6 cystein residues that form three intramolecular disufide bonds [16]. Other well-known members of the 6-cys family are e.g. Pfs 48/45, Pfs230, Pf12 [16].

Peptides of Pf38 were shown to have a high affinity for erythrocytes and diminished their invasion by Plasmodium [17]. Furthermore, Pf38 is well-recognised by sera from adults from Papua New Guinea [14] and Kenya [18] and has been identified as one of few Plasmodium surface proteins to have a signature of balancing selection [19], [20] (i.e., polymorphisms of Pf38 are maintained to facilitate immunoevasion by the parasite). Finally, because Pf38 is expressed during both the asexual and sexual stages [14], blocking Pf38 could both prevent the blood-stage symptoms of malaria and block transmission of the disease, which makes Pf38 an interesting potential malaria antigen. As a production platform we used transiently transformed *Nicotiana benthamiana*. Although the production of recombinant proteins in plants is slightly unusual this system has worked well for us in the past; it has also been used successfully for the production of other malaria vaccine candidates [21], [22]. In order to investigate the suitability of Pf38 as a malaria vaccine, we produced Pf38 and a fusion protein composed of red fluorescent protein and Pf38 (RFP-Pf38). Both recombinant proteins were used for generation of polyclonal antibodies which proved to have an inhibitory effect on the development of the malaria parasite.

## Results

### Production and purification of Pf38 and RFP-Pf38

**Figure 1:**
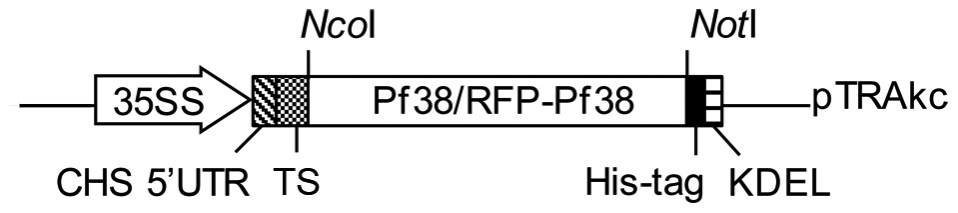
Pf38 and RFP-Pf38 plant expression cassette. 35SS: 35S promoter of the *Cauliflower mosaic virus* with duplicated 35S enhancer region; CHS 5′ UTR: 5′ untranslated region of chalcone synthase (*Petroselinum crispum*); TS: Targeting signal sequence; RFP-Pf38: cDNA encoding the fusion protein comprising a red fluorescent protein and Pf38 of *P. falciparum*; Pf38: cDNA encoding protein Pf38 of *P. falciparum*; His-tag: sequence coding for six histidines; KDEL: retention signal for the endoplasmatic reticulum; *Nco*I/*Not*I: restriction sites used for cloning; pTRAkc: vector backbone of pTRAkc.

**Figure 2:**
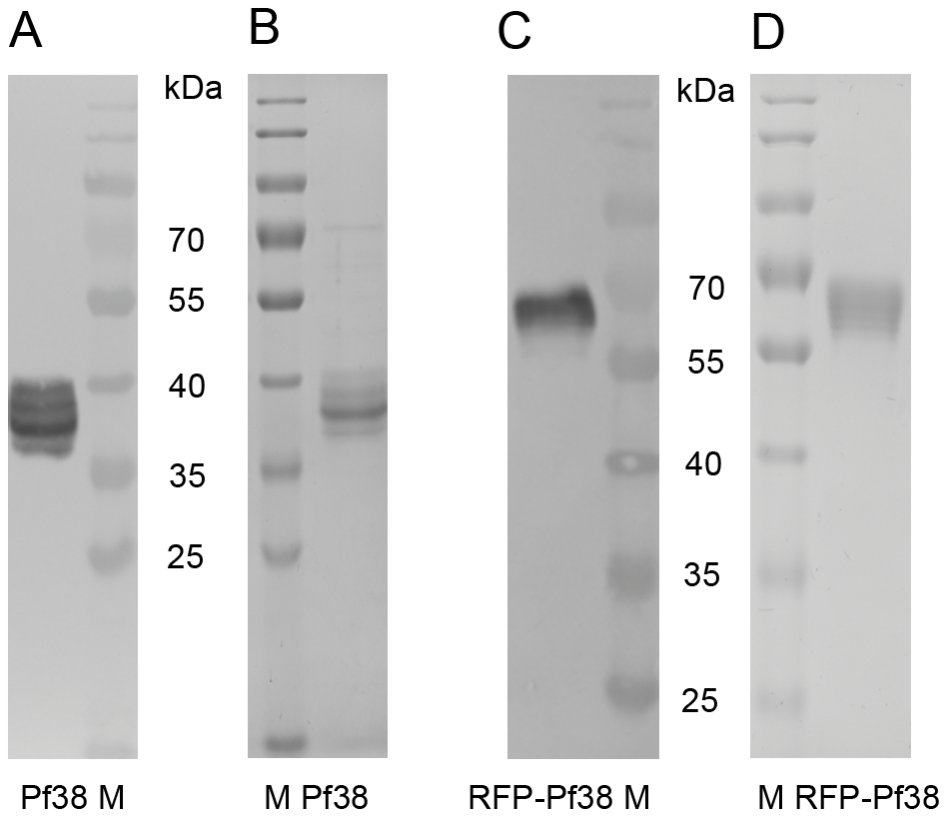
Purity and integrity of Pf38 and RFP-Pf38. Coomassie-stained SDS-PAGE (B, D) and immunoblot (A, C) analysis of Pf38 (A, B) and RFP-Pf38 (C, D) after purification. Detection of recombinant proteins was performed using a rabbit αHis antiserum and an alkaline phosphatase-labelled goat αrabbit antiserum with nitroblue tetrazolium/5-bromo-4-chloro-3-indolyl-phosphate solution as the substrate. M: PageRuler™ Prestained protein ladder (Fermentas), Pf38: 1 µg of Pf38, RFP-Pf38: 1 µg RFP-Pf38.

The DNA-sequences coding for Pf38 and RFP-Pf38 were cloned into the plant expression vector pTRAkc [23]. A schematic depiction of the expression cassette is shown in Figure 1. *Agrobacterium tumefaciens* was transformed with either of the two vectors; cultures of these transgenic *A. tumefaciens* were used to transiently transform *N. benthamiana*. After a few days of incubation the leaves of the transiently transformed plants were harvested. Pf38 and RFP-Pf38 were both successfully produced in and purified from these transiently transformed *N. benthamiana* leaves, although the yield of the recombinant proteins was low. Following purification of the proteins, including fractionated ammonium sulphate precipitation, IMAC and anion exchange chromatography, the yield was 4 µg/g leaf fresh weight for Pf38 and 12 µg/g leaf fresh weight for RFP-Pf38, the total amount of Pf38 and RFP-Pf38 purified was 550 µg and 320 µg respectively. The purity and integrity of both recombinant proteins were analysed by SDS-PAGE with subsequent Coomassie staining and immunoblotting (Figure 2). The observed size of the proteins was in accordance with the expected values at 40 kDa (Pf38) and 67 kDa (RFP-Pf38), though some heterogeneities were observed for Pf38. These are probably due to glycosylation as there are three potential glycosylation sites in the sequence of Pf38 (GenBank accession KC987075). Purified samples of Pf38 and RFP-Pf38 were not stable when stored at 4°C (data not shown), and were therefore stored at −80°C until further use.

### Plasma reactivities of African serum samples against Pf38

**Figure 3:**
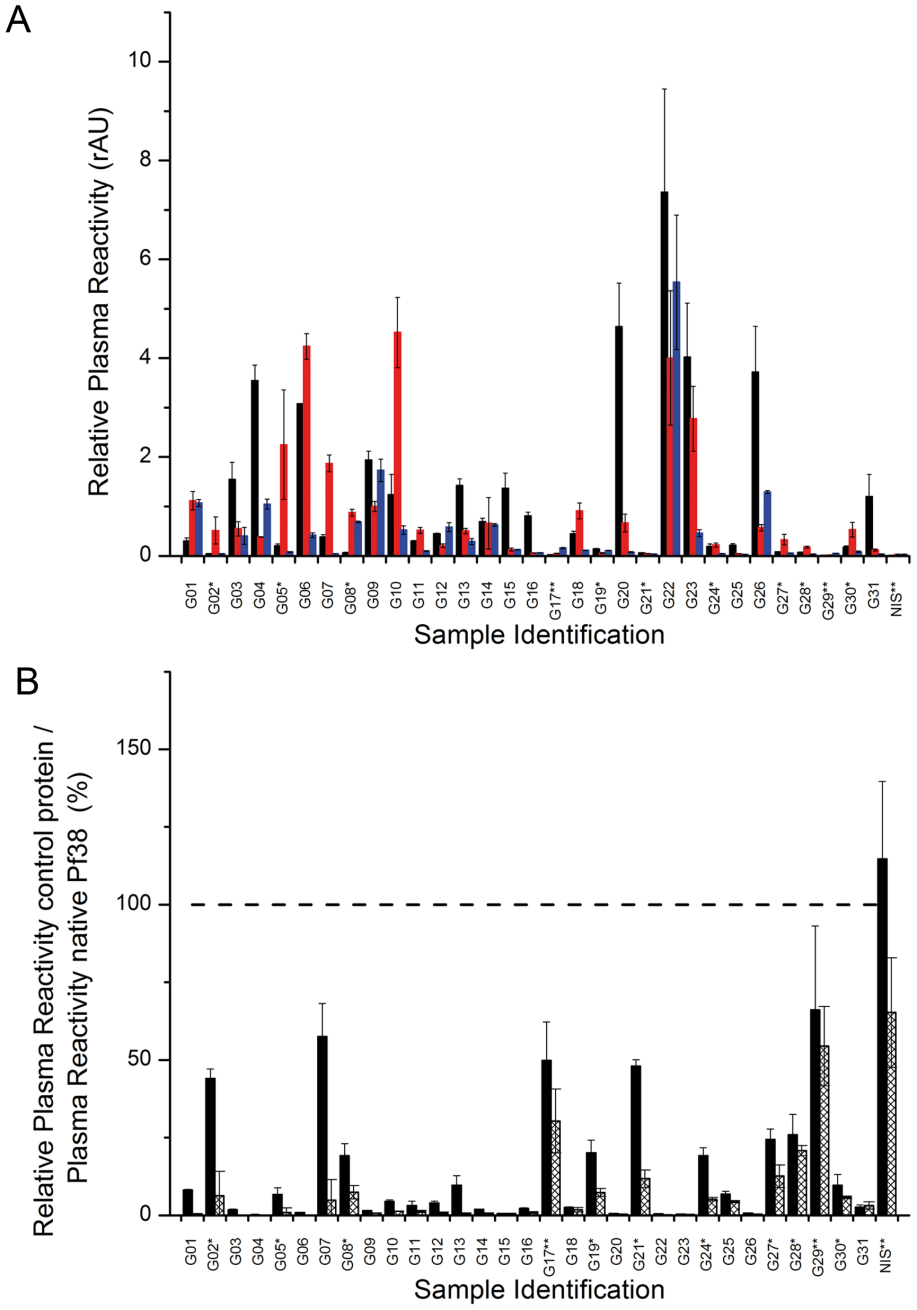
Plasma reactivity of a semi-immune human cohort from Ghana against Pf38 and the control proteins AMA-1, MSP1_19_-His, TFF3-His and denatured PF38. Plasma from 31 semi-immune African serum donors and two European control donors was assayed for reactivity against Pf38 by ELISA. (A) Plasma reactivity is shown as the reactivity of African individual samples (G01–G31) and the European non-immune sample (NIS) relative to a standard African Plasma Pool expressed as relative Arbitrary Units (rAU), +/− the standard deviation. Individual plasma reactivity towards Pf38 (black), AMA-1 (red) and MSP1_19_-His (blue) is shown. (B): Plasma reactivities to denatured and reduced Pf38 (black bars) and TFF3-His (checked bars) are represented as percentage +/− the standard deviation relative to the plasma reactivity to native Pf38. The dashed line represents the reactivity to native Pf38. Check marks at sample Identifications highlight either low (*) or no (**) reactivity of the plasma sample to Pf38. All experiments were performed in triplicate.

Plasma from 31 semi-immune African blood donors was assayed for reactivity against the antigens Pf38, AMA-1 and MSP1_19_-His using an enzyme-linked immunosorbent assay (ELISA). Positive reactivity was defined as a reading of greater than twofold the values obtained for the negative control samples (European serum samples, NIS). The enzymatic immunoabsorbent assay revealed that 94% (corresponding to 29 out of 31 samples) of the African serum samples from semi-immune donors reacted positively against Pf38. A high reactivity (more than twenty fold the negative control reactivity) was found in 61% of the samples, while 35% (11 samples) showed reactivities that were more than one hundred-fold above the negative control reactivity (Figure 3A). The semi-immunity of the African blood donors was confirmed by their reactivity towards two further antigens AMA-1 and MSP1_19_-His (Figure 3A).

The recombinant Pf38 is a His-tagged protein; to control for human serum antibodies binding to the His-tag or to denatured protein, two control ELISA were performed against denatured and reduced Pf38 and against the His-tagged recombinant human protein Trefoil factor 3 (TFF3-His). Denaturing and reducing the protein Pf38 reduced the reactivity by more than 50% in 29 of 31 plasma samples, in more than 67% of the plasma samples, the reactivity was reduced by more than 90%. The reactivity of the plasma samples to the His-tagged control protein TFF3-His is significantly lower than the reactivity to native Pf38 (p<0.0001, Kruskal-Wallis rank sum test). Nevertheless, two samples reached levels of more than 30% reactivity compared to Pf38, but these two samples are both having a reactivity against the antigen Pf38 which is below two-fold the negative control and thus was not regarded as a positive reaction to the target antigen.

### Immunisation and titre determination

The immunogenicity of Pf38 and RFP-Pf38 in BALB/c mice was evaluated using a direct ELISA. Pf38-immunised mice had an average titre of 1∶11.000 against Pf38. RFP-Pf38-immunised mice had an average titre of 1∶560.000 against RFP-Pf38, although titres against Pf38 from these same animals were much lower, with an average titre of 1∶39.000. The sera of the three mice that were immunised with the same antigen were pooled, and the IgGs were purified from both pools via protein G immunoaffinity chromatography. The total amount of IgG after purification was 4.9 mg for Pf38-immunised mice and 3.7 mg for RFP-Pf38-immunised mice.

### Immunofluorescence assay (IFA)

**Figure 4:**
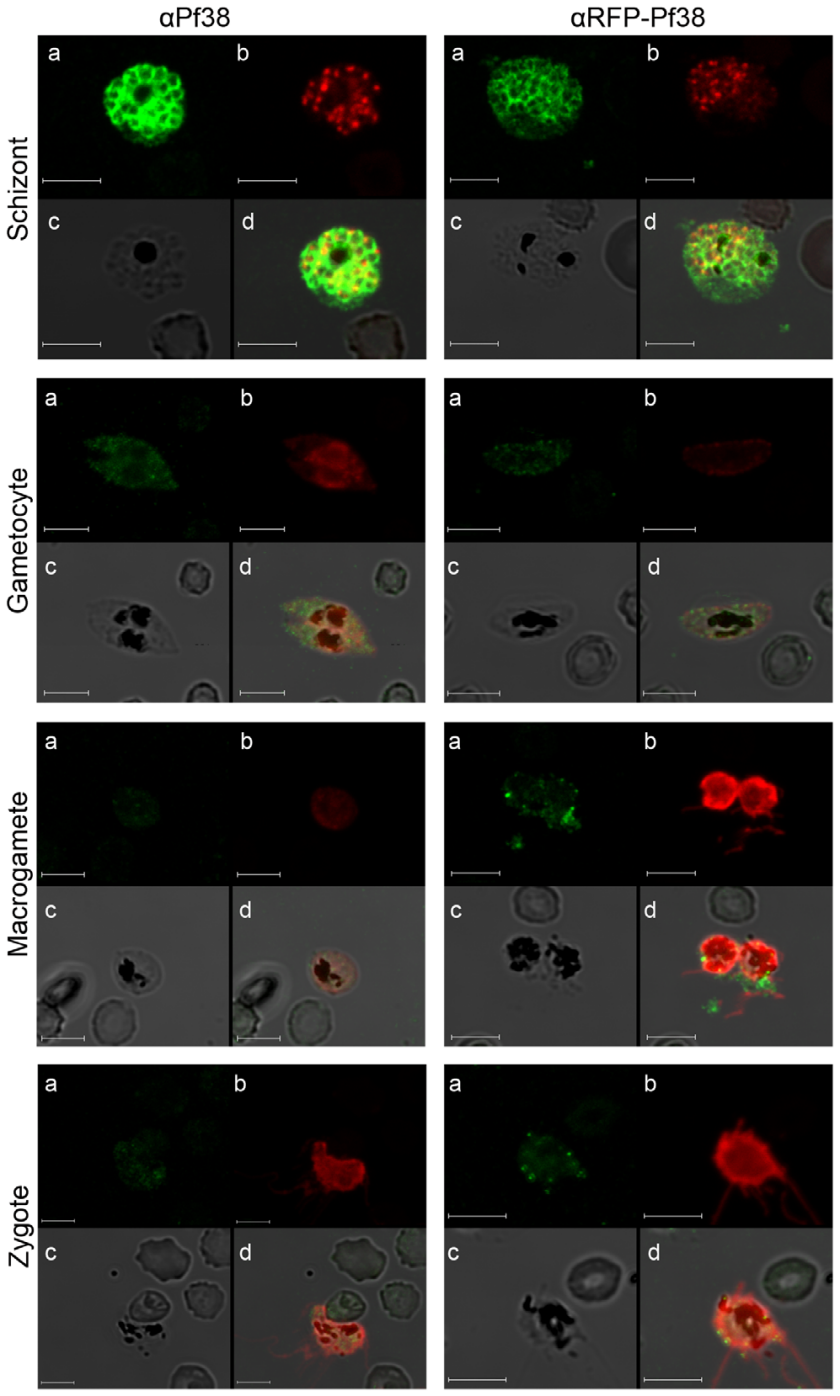
Immunofluorescence assay of NF54 parasites in different stages. For IFAs, *P. falciparum* NF54 parasites in the schizont, gametocyte, macrogamete and zygote stages were fixed with methanol on the surface of a slide. αPf38: Detection was performed using the protein G-purified αPf38 murine IgG fraction. αRFP-Pf38: Detection was performed using the protein G-purified αRFP-Pf38 murine IgG fraction. As a positive control, a rabbit αAMA-1 IgG fraction was used for schizonts, and rabbit αPfs25 serum was used for the sexual stages. (a) Visualisation of murine IgG with Alexa Fluor 488 secondary antibodies (green), (b) visualisation of rabbit IgG with Alexa Fluor 594 secondary antibodies (red), (c) bright light (d) overlay of pictures a, b, and c. Bar: 5 µm.

IFAs were prepared using different lifecycle stages of *P. falciparum*. Mature schizonts were used to check for the presence of Pf38 on the surface of asexual-stage parasites. Strong fluorescence was observed using both αPf38 and αRFP-Pf38 murine IgGs, and this fluorescence coincided clearly with that of control αAMA-1 antibodies (Figure 4, Figure S1). Earlier stages (i.e. ring stage parasites, trophozoites) show weak staining (Figure S2). Pf38 staining on asexual-stage parasites showed an equal distribution of the protein, in accordance with earlier reports [14], while AMA-1 staining was detected only at the apical pole of the developing merozoites [24]. IFA staining of sexual-stage parasites, macrogametes and zygotes produced weak signals, while the gametocyte staining was clearly visible (Figure 4, Figure S1). All Plasmodium lifecycle stages were also treated with a control IgG fraction as a negative control, and no binding could be detected in any of the stages (Figure S3). The specificity of the generated αPf38 and αRFP-Pf38 antibodies was verified by immunoblot analysis of a complete parasite cycle under non-reducing conditions (Text S1, Figure S4).

### Growth inhibition assay (GIA)

**Figure 5:**
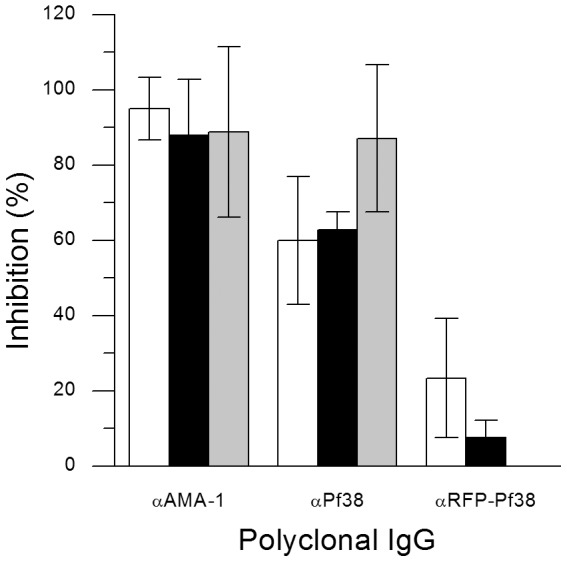
*In vitro* growth inhibition of 3D7A parasites with αPf38 antibodies. A GIA was performed with *P. falciparum* 3D7A parasites and protein G-purified IgG fractions. αAMA-1: Rabbit αAMA-1 IgG fraction (positive control); αPf38: murine αPf38 IgG fraction; αRFP-Pf38: murine αRFP-Pf38 IgG fraction. Each experiment (represented by one bar in the graph) was performed in triplicate. Bars depicted in the same colour indicate experiments that were performed in parallel. Inhibition was calculated as described in the materials and methods.

To evaluate the inhibitory potential of antibodies generated by immunisation with Pf38 or RFP-Pf38 on the invasion of Plasmodium into red blood cells, a GIA was performed on *P. falciparum* 3D7A using the IgG fraction of serum collected from immunised mice (Figure 5). αPf38, αRFP-Pf38 and murine IgG from mice immunised with a non-malaria-related protein (negative control) were tested at a final concentration of 4 mg/ml, while rabbit αAMA-1 (positive control) was used at 6 mg/ml. The negative control was used to calculate the maximal growth of the parasite culture. The positive control demonstrated the expected growth inhibition of 80–100%. In the first two experiments, αPf38 IgG caused an inhibition of approximately 60%, while an inhibition of 87% was achieved in the third experiment. For the αRFP-Pf38 IgG, a growth inhibition of approximately 10–20% was observed for two experiments, while the third experiment failed.

### Zygote development assay (ZDA) and transmission blocking assay (TBA)

ZDA was performed with active and heat-inactivated human sera to investigate the ability of purified αPf38 and αRFP-Pf38 antibodies to initiate complement-mediated lysis of round forms and thus prevent zygote formation. The ZDA (Table 1) showed a significant inhibition equal to 76% for αRFP-Pf38 sera and 74% for αPf38 sera using active human serum. For inactive human serum, no inhibition was detected (Table 2). Four membrane feed experiments were performed on mosquitoes to evaluate the transmission-blocking ability of αPf38 and αRFP-Pf38 antibodies. A mixture of purified gametocytes and fresh erythrocytes was mixed with active human serum supplemented with the appropriate test IgG. The mixture was then fed to mosquitoes, and the guts of these mosquitoes were observed for infection 9–11 days later. Data obtained from the dissected/infected mosquitoes and the counted oocysts (Table 3) demonstrated a significant transmission-blocking activity only in one TBA experiment for the αPf38 antibodies. No transmission-blocking effect has been observed for the αRFP-Pf38 antibodies.

**Table 1:** Results from ZDAs performed in the presence of active human serum.

	Experiment 1		Experiment 2		Experiment 3	
IgG(a)	round forms(b) [%]	P-values(c)	round forms(b) [%]	P-values(c)	round forms(b) [%]	P-values(c)
Control	100±12		100±21		100±10	
RFP-Pf38	26±8	0.0011	21±2	0.0025	25±2	0.0002
Pf38	29±9	0.0016	26±6	0.0035	22±8	0.0004

(a) Protein G-purified IgG from mice were used at a final concentration of 1 mg/ml. Polyclonal IgG purified from mice immunised with a non-malaria-related antigen was used as a negative control (Control). The IgG used originated from mice immunised either with the RFP-Pf38 fusionprotein or with Pf38 alone.

(b) Counts of round forms are given in percentages, and the mean number of round forms in controls was defined as 100%. Means and standard deviations were calculated from the counts of three 0.1 µl aliquots of each sample using a hemocytometer.

(c) Student's t-test was used to statistically analyse the difference in counts of round forms between samples supplemented either with control IgG or test IgG.

P-values below 0.05 were considered significant.

**Table 2:** Results from ZDAs performed in the presence of inactive human serum.

	Experiment 1		Experiment 2		Experiment 3	
IgG(a)	round forms(b) [%]	P-values(c)	round forms(b) [%]	P-values(c)	round forms(b) [%]	P-values(c)
Control	100±30		100±21		100±5	
RFP-Pf38	90±35	0.7183	119±15	0.274	98±11	0.7559
Pf38	125±30	0.3169	115±15	0.356	100±7	0.9488

(a) Protein G-purified IgG from mice were used at a final concentration of 1 mg/ml. Polyclonal IgG purified from mice immunised with a non-malaria-related antigen was used as a negative control (Control). The IgG used originated from mice immunised either with the RFP-Pf38 fusionprotein or with Pf38 alone.

(b) Counts of round forms are given in percentages, and the mean number of round forms in controls was defined as 100%. Means and standard deviations were calculated from the counts of three 0.1 µl aliquots of each sample using a hemocytometer.

(c) Student's t-test was used to statistically analyse the difference in counts of round forms between samples supplemented either with control IgG or test IgG.

P-values below 0.05 were considered significant.

**Table 3:** Evaluation of transmission-blocking activity of purified mouse IgG.

Experiment	IgG(a)	mean number of oocysts (range)	p-values(b)	Mosquitoes infected/dissected	Inhibition of transmission [%]	p-values(c)
1	Control	7 (0–35)		17/20		
	RFP-Pf38	4 (0–33)	0.258	15/20	12	0.6948
	Pf38	9 (0–35)	0.758	13/19	20	0.2733
2	Control	7 (0–28)		17/20		
	RFP-Pf*3*8	7 (0–28)	0.779	16/20	6	0.4075
	Pf38	1 (0–13)	0.01	9/20	47	0.0187
3	Control	2 (0–12)		13/20		
	RFP-Pf38	6 (0–39)	0.242	15/20	0	0.7311
	Pf38	7 (0–37)	0.231	15/20	0	0.7311
4	Control	1 (0–7)		7/20		
	RFP-Pf38	1 (0–2)	0.947	8/20	0	>0.9999
	Pf38	2 (0–19)	0.841	7/20	0	>0.9999

(a) Protein G-purified polyclonal IgG from mice were used at a final concentration of 1 mg/ml. Polyclonal IgG purified from mice immunised with a non-malaria-related antigen was used as a negative control (Control). The antisera used originated from mice either immunised with the RFP-Pf38 fusionprotein or with Pf38 alone.

(b) A Mann-Whitney U test was used to analyse the median numbers of oocysts between groups of mosquitoes either receiving control IgG or test IgG during infection with *P. falciparum*. P-values below 0.05 were considered significant.

(c) Fisher's exact test was used to analyse the oocyst prevalence between groups of mosquitoes either receiving control IgG or test IgG during infection with *P. falciparum*.

P-values below 0.05 were considered significant.

## Discussion

Pf38 has been suggested as an interesting candidate for a malaria vaccine [14], [17]. To investigate this possibility, we produced both Pf38 and a RFP-Pf38 fusion protein. Plants were used for the production of recombinant Pf38 and RFP-Pf38, as plants offer some benefits over other production systems, such as a fast appraisal of constructs [25] and cost-effective large-scale production [26]. Moreover, plant-derived proteins are free of endotoxins and human pathogens [27]. While other reports on Pf38 exist [14], [15]–[17], [18], this study is the first to report that recombinant Pf38 can be produced and purified. Although our yields of Pf38 and RFP-Pf38 after purification were relatively low (4 µg and 12 µg per gram leaf fresh weight, respectively), especially when compared to other *P. falciparum* proteins produced in *N. benthamiana* [21], [22], the amounts of Pf38 and RFP-Pf38 generated were sufficient for immunisations and screenings. The quality of the recombinant Pf38 was assessed by ELISA using serum samples from malaria semi-immune donors, and over ninety percent of the tested serum samples reacted positively or very positively with the native recombinant Pf38. (Figure 3A). In contrast to this, antibodies from semi-immune donors did not react strongly to denatured and reduced recombinant Pf38 (Figure 3B). This suggests a near native conformation of the plant-produced Pf38. Additionally, the presence of these antibodies in the blood-donors that were recruited on the basis of being free from clinical malaria infection for at least two years, might give a hint for a role for Pf38 in protective immunity. However, the later hypothesis needs to be confirmed in a survey comparing a population protected against clinical malaria vs. a population living in the same setting while not being protected.

We generated the fusion variant of Pf38 for two reasons. First, the expression of RFP has worked well in our lab [28], [29], and we hypothesised that an attached RFP might help stabilise the protein. Second, no prior knowledge existed regarding the immunogenicity of Pf38. Using the Immune Epitope Database and Analysis Resource tool for the prediction of MHC-II T-cell epitopes [30], we found that only moderate affinities for Pf38 were predicted. Therefore, the attachment of RFP, which has high immunogenicity itself, could boost the immunogenic effect of Pf38. This hypothesis proved to be true, as the titres against Pf38 were nearly four times higher in RFP-Pf38-immunised mice (1∶38.000) versus Pf38-immunised mice (1∶11.000); however, the quality of the immune responses was also different, with the IgG fraction purified from the serum of Pf38-immunised mice being more effective (Figure 5; Table 3). The IFAs further confirmed that plant-produced Pf38 was able to induce the generation of specific αPf38 antibodies in mice, which could detect native Pf38 on *P. falciparum* surfaces in different stages (Figure 4). While Pf38 has been detected on schizonts [18], this is to our knowledge the first study to report the detection of Pf38 on the surface of gametocytes, macrogametes and zygotes. The IFA results also confirmed previous transcription data suggesting that Pf38 is present on the surface of schizonts and gametocytes [14], [15]. Although it has been predicted that Pf38 is present on the surface of sporozoites as well [14], [15], it was not possible to identify the protein on sporozoites by IFA here (data not shown).

To evaluate the inhibitory functions of αPf38 and αRFP-PF38 antibodies, we first focused on the blood stages of the parasite. The inhibition of erythrocyte invasion by merozoites was analysed using a GIA, and the *in vitro* growth inhibition of *P. falciparum* 3D7A with αPf38 antibodies showed a 60% to 87% inhibition. In comparison to αAMA-1 antibodies, which were used as a control (demonstrating 80–100% inhibition), αPf38 antibodies showed relatively strong inhibition; moreover, these high inhibitory values relative to AMA-1 were obtained despite the amount of αAMA-1 antibodies being 50% greater than the amount of αPf38 antibodies used in the assay (an adjustment that was made to compensate for the relative small amount of serum that could be obtained from mice). Data on a GIA using αPf38 antibodies from rabbits obtained via a viral vectored vaccine [18] show a lower inhibition, which might be due to the different experimental approach. Although the titre against Pf38 from RFP-Pf38-immunised mice was approximately four times higher than titres from Pf38-immunised mice, growth inhibition by these antibodies was only 10–20%, suggesting that titre alone is not the deciding factor for strong inhibition. One can speculate that native Pf38 may fold more efficiently than a fusion Pf38 construct, resulting in antibodies whose paratopes are better suited for recognition of native Pf38.

As Pf38 was detected on the surface of sexual-stage *P. falciparum*, the ability of αPf38 and αRFP-Pf38 antibodies to inhibit parasite round forms and thus prevent zygote formation was assessed. Our results (Table 1) demonstrated significant inhibitions of 76% and 74% with αRFP-Pf38 and αPf38 antibodies, respectively, in the presence of active human serum. In the presence of inactive sera, no significant inhibition could be achieved. By using the round-form inhibition assay with active and inactive sera, insights into the mode of inhibition could be gained. Because active human serum was necessary for inhibition, it is likely that activation of the complement system, similar to that described for Pfs230 [31], is a crucial part of the observed inhibition. As zygote formation was strongly inhibited, it was of interest to study whether the αPf38 and αRFP-Pf38 antibodies would also have transmission-blocking ability. Only one out of four experiments showed a significant inhibition of transmission by αPf38 antibodies, which was unexpected. It can be assumed that the amount of antibodies reaching the mosquito gut in the TBA is more variable in comparison to the amount of antibodies used in the ZDA, as factors such as the size of the blood meal and the individual mosquito gut environment will influence the concentration of active antibodies in the gut. Future experiments would benefit from the use of greater concentrations of αPf38 antibodies in TBAs i.e. an optimisation of immunisation strategies. Currently, it should be noted that no significant transmission-blocking activity was observed.

In summary, recombinant Pf38 is able to induce an immune response in mice, and the murine antibodies generated during this response can strongly inhibit the growth of blood-stage *P. falciparum* and the formation of round forms *in vitro*. Furthermore the strong reactivities of semi-immune African donors to Pf38 give an indication of the involvement of αPf38 antibodies in protective immunity against malaria. We agree that an effective malaria vaccine should most likely be based on several antigens [32] or subdomains directed to several stages of the parasite lifecycle [17]; Pf38 or a part of Pf38 could be one such subdomain of a malaria vaccine. Unfortunately, the yields of recombinant Pf38 in plants were low, although this problem may be alleviated by producing only one part of Pf38 or by producing Pf38 in the context of a larger construct consisting of various malaria-related antigens. From the observations made in this study, we conclude that Pf38 is a promising candidate for the development of a multi-stage malaria vaccine.

## Materials and Methods

### Ethics statement

The animal experiments were officially approved by the Landesamt für Natur, Umwelt und Verbraucherschutz Nordrhein-Westfalen (LANUV), reference number 8.87.-51.05.30.10.077. All animals received humane care in accordance with the requirements of the German Tierschutzgesetz, §8 Abs. 1 and in accordance with the Guide for the Care and Use of Laboratory Animals published by the National Institute of Health.

The human study was conducted according to the Helsinki Declaration on Scientific Research and received approval from the regional Committee on Human Research Publication and Ethics (CHRPE) of The Kwame Nkrumah University of Science and Technology. After explaining the aims and procedures of the study, written informed consent was obtained from the participants. All study participants were clinically examined and all pregnant or nursing mothers, as well as anaemic volunteers, were excluded from the study.

### Bacteria, plants and parasites

*Agrobacterium tumefaciens* strain GV3101 : : pMP90RK [GmR, KmR, RifR] [33] and *N. benthamiana* plants were used for agroinfiltration, i.e., the production of recombinant proteins by plants. For IFA, ZDA and TBA, the parasite *P. falciparum* NF54 (MRA-1000, MR4, Manassas, USA) was used, while GIA were performed using *P. falciparum* 3D7A (MRA-151, MR4, Manassas, USA), a substrain of NF54 [34].

### Plant expression vector design and construction

The cDNA for Pf38 was constructed based on the Pf38 cDNA from *P. falciparum* (GenBank accession ABO41470.1). The DNA sequence encoding the last 21 amino acids, which form the GPI anchor attachment region, was omitted, and the remainder of the gene was codon optimised for tobacco. The following short sequence additions were made at either ends of the cDNA to facilitate cloning: 5′ GGTACCGAATTCTCCATGGTGGCGGCCGGAGACCTGCGTATT 3′ at the 5′ end of the gene, adding a *Nco*I and a *Bsa*I restriction site; and 5′ CGGCCGCGAGGCATAGGGATCCTCTAGATGGAGCT 3′ at the 3′ end of the gene, adding a *Not*I, *Bam*HI and *Xba*I restriction site. The final sequence (GenBank accession KC987075) was used to produce a synthetic gene (GeneArt), and the gene was cloned via *Nco*I/*Not*I into pTRAkc-ERH and pTRAkc-rfp-ERH [23]. Both vectors contained the DNA sequence for a signal peptid, a His-tag and a retention signal for the endoplasmatic reticulum, pTRAkc-rfp-ERH contains also the cDNA for a tetrameric red fluorescent protein (RFP) of *Discosoma spec*. (Figure 1). All cloning was confirmed by sequencing.

For the silencing inhibitor, the p19 gene was amplified by PCR from pEAQ-HT [35] using primers to introduce an *Nco*I site at the start codon and a *Spe*I site behind the stop codon. The *Nco*I/*Spe*I-digested PCR product was ligated into *Afl*III/*Xba*I-digested pTRAc to produce pTRAc-p19si.

### Transient transformation of *N. benthamiana*

All three plant expression vectors described above were separately introduced into *A. tumefaciens* using a Multiporator (Eppendorf AG), according to the manufacturer's instructions.

*A. tumefaciens* cells containing one of the three plasmids (pTRAc-p19si, pTRAkc-Pf38-ERH or pTRAkc-RFP-Pf38-ERH) were cultivated in YEB medium [36] containing 50 µg/ml carbenicillin, 25 µg/ml kanamycin and 25 µg/ml rifampicin. One day before the infiltration, the cultures were pre-induced by the addition of 2-(*N*-morpholino)ethanesulfonic acid from a stock solution (pH 5.6), glucose (to a final concentration of 10 mM) and acetosyringone (20 µM final concentration). On the day of the infiltration, the cultures were prepared by dilution to an OD_600_ = 2 using water. The diluted cultures were then mixed in equal parts with 2× infiltration medium (10% (w/v) sucrose, 0.398% (w/v) glucose, 0.01% (w/v) Ferty2 Mega (Planta Düngemittel GmbH, pH 5.6) and acetosyringone (200 µM final concentration) and incubated for 2 hours at room temperature. The final infiltration suspension contained 80% (v/v) of the induced agrobacteria containing pTRAkc-Pf38-ERH (or pTRAkc-rfp-Pf38-ERH) and 20% (v/v) of the induced agrobacteria containing pTRAc-p19si. Plants used for agroinfiltration were grown in a greenhouse under a 16 hours natural daylight photoperiod and a 25°C/day and 22°C/night temperature regimen. The plants were approximately eight weeks old at the time of infiltration.

Whole *N. benthamiana* plants with roots in rock wool were immersed upside down into the infiltration suspension and were vacuum-infiltrated [23]. After the infiltration, the plants were cultivated for 4–10 days at 25°C with 16 hours of light per day (110 µE) and regular watering. Only leaves were harvested and stored at −20°C until later use.

### Purification of Pf38 and RFP-Pf38

For the purification of Pf38, the total soluble protein from infiltrated tobacco leaves was extracted by macerating a 1∶3 (w/v) ratio of plant material and ice cold extraction buffer (100 mM Tris-HCl pH 8, 100 mM NaCl, 10 mM sodium disulphite, 10 mM EDTA, 2 mM DTT, 10 mM benzamidine, 5 mM aminocaproic acid, 5 mM imidazole) in a blender. The resulting green slurry was first filtered through a double layer of Miracloth and then mixed with solid ammonium sulphate to 30% saturation. After centrifugation at 40.000 g (4°C, 20 minutes), the supernatant was separated from the pellet and then mixed with ammonium sulphate to a saturation of 60%. The sample was then centrifuged as described above. The supernatant was discarded, and the pellet was redissolved in NTA-buffer (50 mM Tris-HCl pH 8, 500 mM NaCl) containing 5 mM imidazole and applied to a Ni-NTA matrix (Macherey-Nagel). The column was washed with 40 column volumes (cv) washing buffer (NTA-buffer containing 10 mM imidazole). Elution of the recombinant proteins from the Ni-NTA was achieved with elution buffer (NTA-buffer containing 200 mM imidazole). The elution fractions were tested via SDS-PAGE and immunoblot for the presence of Pf38, and the fractions that contained the recombinant proteins were combined. A buffer exchange into AEX-buffer (20 mM sodium phosphate pH 6.9) was performed using PD10-columns (GE Healthcare). Anion exchange chromatography was carried out using DEAE sepharose fast flow (GE Healthcare Bio-Sciences AB). The sample was loaded onto the column, and the flowthrough was collected. The column was then washed with 4 cv AEX buffer containing 20 mM NaCl, and the flowthrough and the wash fraction were combined, buffer exchanged into PBS and subsequently concentrated using centrifugal concentrators (Vivaspin 6, MWCO 10 kDa; Sartorius Stedim Biotech GmbH). Until further use, the purified proteins were stored at −80°C. RFP-PF38 was purified in the same way, except that no imidazole was added to the extraction buffer or NTA-buffer.

Analysis of the purity and integrity of the purified proteins was performed via SDS-PAGE with subsequent Coomassie staining and immunoblotting. This was done as described before [37], and Pf38 was detected via the His-tag attached to the protein using a rabbit αHis antiserum (GenScript). For the secondary antibody, an alkaline phosphatase-labelled goat αrabbit antiserum (Jackson ImmunoResearch) was used, and nitroblue tetrazolium/5-bromo-4-chloro-3-indolyl-phosphate solution was utilised as the substrate.

### Plasma reactivities of African serum samples against Pf38

From 31 semi-immune adults from the region of Kumasi, Ghana, 7.5 ml of blood was withdrawn into heparinised blood tubes. Blood donors were selected on the basis of living in this holoendemic region and being free from clinical malaria infection for at least two years. Plasma was separated and frozen until it was used in an ELISA. As a negative control, plasma from two European donors, who had never experienced malarial infection, was withdrawn and treated in the same way.

To investigate naturally occurring reactivity against the Pf38 antigen, an ELISA was performed according to standardised procedures with small modifications [38], [39]. Besides Pf38, two positive controls, AMA-1 (diversity covering PfAMA-1 produced in *Pichia pastoris*) [40] and Msp1_19_-His (3D7; produced in HEK293) and an unrelated His-tagged protein, the human Trefoil factor 3 (TFF3-His, produced in Hek293) as a negative control were tested in the same ELISA. In short, 50 ng of antigen at a concentration of 1 µg/ml was coated overnight on 96-well high-binding EIA plates in coating buffer (0.1 M sodium carbonate). To prevent unspecific binding, wells were blocked with PBS containing 2% (w/v) BSA. Human plasma samples were added at serial dilutions. As a positive control, a pool of plasma from semi-immune adults was also prepared and added at serial dilutions. Human IgGs were detected via an alkaline phosphate-conjugated goat αhuman IgG (Jackson ImmunoResearch) and subsequent hydrolysation of pNPP. The absorbance was measured at 405 nm, and the data were analysed using the R language for statistical computing v2.13 [41].

In order to compare the reactivity of human antibodies from semi-immune blood donors to native and denatured Pf38, an ELISA was performed on native Pf38 and reduced and denatured Pf38. Denaturing of the antigen was performed in 4 M Urea and 10 mM DTT at 56°C for 30 min at an antigen concentration of 10 µg/ml. Subsequently, the denatured antigen was coated on EIA plates in a coating buffer (0.1 M sodium carbonate) at a concentration of 1 µg/ml and the ELISA was then performed as described above.

### Immunisation of mice and collection of sera

Groups of three BALB/c mice, between 6 and 8 weeks of age (Taconic), were immunised intraperitoneally with 17 µg Pf38 or RFP-Pf38 emulsified in adjuvant Gerbu MM (Gerbu Biotechnik GmbH). Another group of four mice was immunised with a non-malaria-related antigen emulsified in Gerbu MM as a negative control. The primary immunisation contained 40 µl Gerbu MM, with the three subsequent boosts containing 20 µl Gerbu MM each. The immunisations were performed every two weeks. Blood from the tail veins of the mice was collected at the same time that the immunisations were performed to check for titre development. Fourteen days after the last immunisation, the mice were narcotised, and blood was collected by cardiocentesis. After a 20 minutes incubation at RT, the blood was centrifuged (1000 g for 10 minutes at 4°C), and the serum was separated from the blood cells, mixed with 0.05% (w/v) sodium azide and kept at 4°C until further use.

### Determination of titres of murine αPf38 and αRFP-Pf38

The titers were determined with a standard direct ELISA. 100 ng Pf38 and RFP-Pf38/well were coated in PBS. As primary antibodies serial diluted mouse serum was used at a dilution range of 1∶500–1∶1.024.000. The secondary antibody was goat αmouse IgG (Fcγ) labelled with horseradish peroxidase (Jackson ImmunoResearch). As substrate 2, 2′-azino-bis(3-ethylbenzothiazoline-6-sulphonic acid) (Roche) with the appropriate buffer was used according to the manufacturers manual and absorbance was read at 405 nm after 30 minutes. Titres were determined as the dilution that gave the double value of the background (pre-immune serum).

### Preparation of mouse sera

Total IgG was purified from pooled sera obtained from immunised mice or control mice using a protein G-Sepharose column (GE Healthcare) according to the manufacturer's instructions. The serum samples, after a 10-fold dilution with binding buffer (50 mM acetate buffer, pH 5.0), were loaded onto a protein G-Sepharose column pre-equilibrated with binding buffer. The column was washed with 10 cv of binding buffer. Bound IgG was eluted with 100 mM glycine-HCl (pH 2.7), and the protein concentration was determined by Bradford assay. The complete eluent was filter-sterilised and concentrated to 8 mg/ml, and the buffer was exchanged to RPMI 1640 using a 30 kDa cutoff in AmiconUltra-15 tubes (Millipore). Finally, the purified IgG fraction was stored at 4°C until further use.

### IFA

The cultivation of parasite asexual and sexual stages and indirect IFA were performed in the main as described previously [42], [43]. For IFA, parasite preparations were air-dried on 8-well diagnostic slides and fixed with −80°C methanol for 10 minutes. To block nonspecific binding and to permeabilise membranes, fixed cells were incubated in blocking solution (0.5% (w/v) BSA, 0.01% (w/v) saponin, 1% (v/v) neutral goat serum in PBS) for 30 minutes at room temperature. The cells were then incubated with either the total IgG fraction derived from Pf38-immunised mice or RFP-Pf38-immunised mice diluted 1/50 in blocking solution without goat serum at 37°C for 1 hour. For visual identification of different *P. falciparum* life cycle stages, specific rabbit-derived antibodies were used. For the visualisation of schizonts, rabbit αAMA-1 (BG98 standard, kindly provided by BPRC, Rijswijk, The Netherlands) was used; for gametocytes, macrogametes and zygotes, rabbit αPfs25 (MRA-38, MR4, Manassas, USA) was used. Visualisation of primary antibodies was achieved by subsequent incubation of cells with fluorescence-conjugated goat αmouse (Alexa Fluor 488) and goat αrabbit (Alexa Fluor 594) antibodies (Invitrogen). If no labelling of parasites using rabbit antibodies was performed, cells were counterstained with 0.05% (w/v) Evans Blue in PBS. Finally, cells were mounted with the anti-fading solution AF2 (Citifluor Ltd.), and the slides were sealed with nail varnish. The examination of labelled cells and image scans were performed using a Leica SP5 confocal microscope (Leica).

### GIA

GIAs were performed as described previously [40]. Briefly, protein G-purified IgG fractions (4 mg/ml final concentration), positive control rabbit αAMA-1 (6 mg/ml final concentration), synchronised *P. falciparum* schizonts and culture medium were applied to half-area 96-well tissue culture plates in a total volume of 50 µl/well. Each sample was tested in triplicate, and the experiment was repeated twice. After an incubation of 40–42 hours, the parasitemia levels were determined using a parasite lactate dehydrogenase assay. The percent inhibition of parasite invasion was calculated as described previously [40].

### ZDA

The ZDA used here was based on a previous publication [44]. The ability of mouse αPf38/αRFP-Pf38 antibodies to initiate complement-mediated lysis of round forms and thus prevent zygote formation was analysed using the round-form inhibition assay. *P. falciparum* cultures containing mature stage V gametocytes showing substantial exflagellation were aliquoted and centrifuged. Cells were resuspended either in active human A^+^-serum or heat-inactivated A^+^-serum supplemented with the respective test IgG. Gametogenesis was induced by the addition of 0.1 mM xanthurenic acid and incubation at room temperature. Samples were incubated overnight. Macrogametes and zygotes were visualised by incubating cells with mouse αPfs25 antibodies, followed by labelling with Alexa Fluor 488-conjugated goat αmouse antibodies (Invitrogen). The counting of round forms was performed using a hemocytometer and a ZEISS Axiolab fluorescence microscope (Zeiss).

### TBA

To assess the ability of mouse αPf38/αRFP-Pf38 antibodies to block the transmission of *P. falciparum* from the human to the mosquito, membrane feeding assays were performed [45]. Briefly, mature stage V gametocytes were first purified by Percoll density gradient centrifugation from cultures showing substantial exflagellation [46] and then mixed with an equal amount of fresh A^+^-erythrocytes. Cells were then mixed with an equal amount of active human A^+^-serum supplemented with the respective test IgG and directly fed to gentamicin-treated *A. stephensi* mosquitoes through a thin layer of parafilm stretched across the bottom of a glass feeder heated to 38°C [47]. The mosquitoes were allowed to feed for 20 minutes and were subsequently kept in a secured insectary at 80% humidity and 26°C. To measure the infectivity of the different samples, 20 midguts of blood-fed mosquitoes were dissected 9–11 days after the infection and stained with 0.2% (v/v) mercurochrome in PBS to facilitate counting of oocysts. A Mann-Whitney U test was used to statistically analyse the median numbers of oocysts between groups of mosquitoes either receiving neutral mouse serum or test sera during infection with *P. falciparum*. Additionally, the oocyst prevalence between groups of mosquitoes either receiving control IgG or test IgG during infection with *P. falciparum* was statistically analysed using Fisher's exact test [48].

## Supporting Information

Figure S1 **Immunofluorescence assay of NF54 parasites in different sexual stages.** For IFAs, *P. falciparum* NF54 parasites in the gametocyte (A), macrogamete (B) and zygote (C) stages were fixed with methanol on the surface of a slide. αPf38: Detection was performed using the protein G-purified αPf38 murine IgG fraction. αRFP-Pf38: Detection was performed using the protein G-purified αRFP-Pf38 murine IgG fraction. As a positive control rabbit αPfs25 serum was used. (a) Visualisation of murine IgG with Alexa Fluor 488 secondary antibodies (green), (b) visualisation of rabbit IgG with Alexa Fluor 594 secondary antibodies (red), (c) bright light (d) overlay of pictures a, b, and c. Bar: 5 µm.
(TIF)

Figure S2 **Immunofluorescence assay of NF54 parasites in ring stage and trophozoite stage.** For IFAs, *P. falciparum* NF54 parasites in ring stage (A) and trophozoite stage (B) were fixed with methanol on the surface of a slide. αPf38: Detection was performed using the protein G-purified αPf38 murine IgG fraction. αRFP-Pf38: Detection was performed using the protein G-purified αRFP-Pf38 murine IgG fraction. NMS: Detection was performed using the protein G-purified murine IgG fraction of neutral mouse serum. As a positive control, a rabbit αAMA-1 IgG fraction was used. (a) Nuclei were stained with Hoechst 33342. (b) Visualisation of murine IgG with Alexa Fluor 488 secondary antibodies (green), (c) visualisation of rabbit IgG with Alexa Fluor 594 secondary antibodies (red), (d) bright light (e) overlay of pictures a, b, c and d. Bar: 5 µm.
(TIF)

Figure S3 **Negative controls for Immunofluorescence assays depicted in** **Figure 4** **and Figure S1.** For IFAs, *P. falciparum* NF54 parasites in the schizont, gametocyte, macrogamete and zygote stages were fixed with methanol on the surface of a slide. Detection was performed using the protein G-purified murine IgG fraction of neutral mouse serum. As a positive control, a rabbit αAMA-1 IgG fraction was used for schizonts, and rabbit αPfs25 serum was used for the sexual stages. (a) Visualisation of murine IgG with Alexa Fluor 488 secondary antibodies (green), (b) visualisation of rabbit IgG with Alexa Fluor 594 secondary antibodies (red), (c) bright light (d) overlay of pictures a, b, and c. Bar: 5 µm.
(TIF)

Figure S4 **Immunoblot to prove specificity of generated αPf38 sera.** Parasite preparations and plant produced Pf38 were run on SDS-PAGE gel and subsequently blotted. Detection of Pf38 was performed using murine αPf38 IgG (A) or murine αRFP-Pf38 IgG (B) and an alkaline phosphatase-labelled goat αmouse antiserum with nitroblue tetrazolium/5-bromo-4-chloro-3-indolyl-phosphate solution as the substrate. For all asexual stages 4.5×10^6^ parasites were used, while the gametocyte preparation contained 5×10^5^ gametocytes. 1: Asexual parasites 12 h after invasion 2: Asexual parasites 24 h after invasion 3: Asexual parasites 36 h after invasion 4: Asexual parasites 48 h after invasion 5: plant produced Pf38 (600 ng) 6: gametocyte preparation M: PageRuler™ Prestained protein ladder (Fermentas).
(TIF)

Text S1 **Preparation and results of immunoblot depicted in Figure S4.**
(DOCX)
