# Supplementary figures and images for: Plant-Based Production of Recombinant Plasmodium Surface Protein Pf38 and Evaluation of its Potential as a Vaccine Candidate
Source: PLoS One. 2013 Nov 21;8(11):e79920. doi: 10.1371/journal.pone.0079920 (PMC3836784; doi:10.1371/journal.pone.0079920)

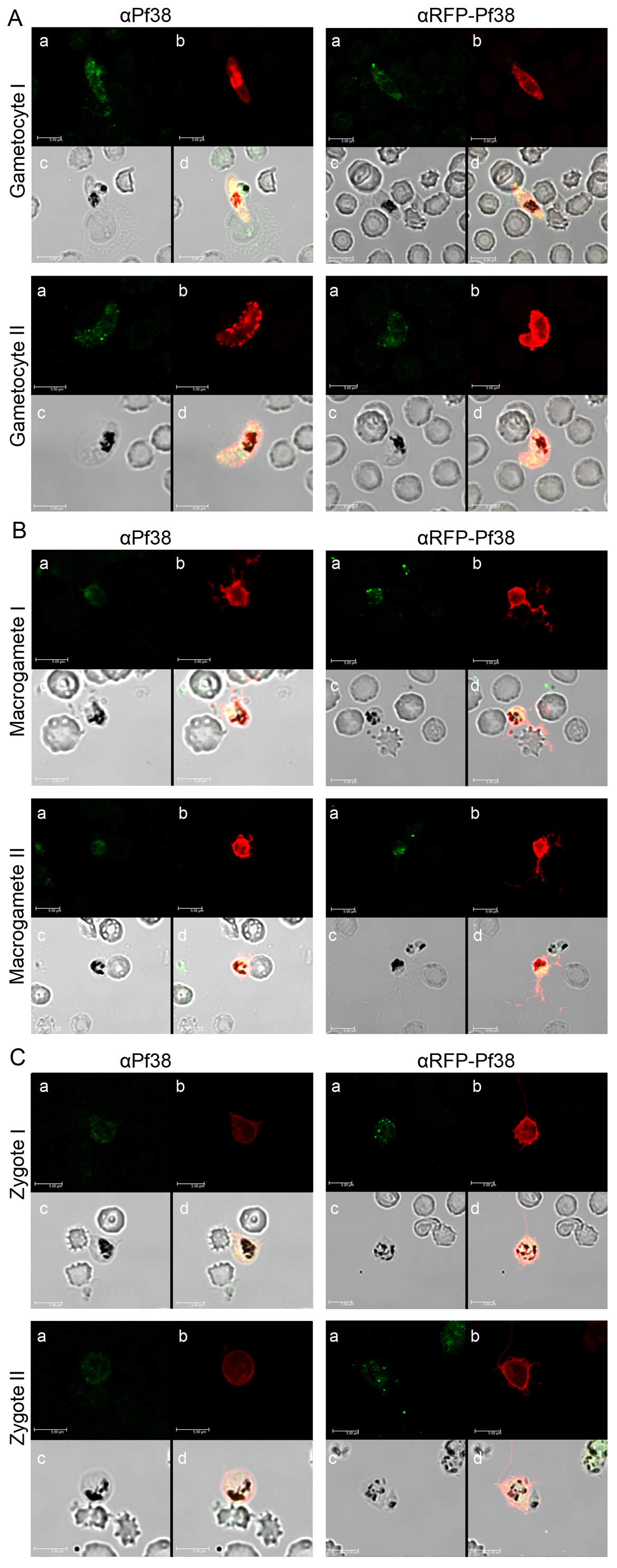

Supplement: Figure S1 — Immunofluorescence assay of NF54 parasites in different sexual stages. For IFAs, P. falciparum NF54 parasites in the gametocyte (A), macrogamete (B) and zygote (C) stages were fixed with methanol on the surface of a slide. αPf38: Detection was performed using the protein G-purified αPf38 murine IgG fraction. αRFP-Pf38: Detection was performed using the protein G-purified αRFP-Pf38 murine IgG fraction. As a positive control rabbit αPfs25 serum was used. (a) Visualisation of murine IgG with Alexa Fluor 488 secondary antibodies (green), (b) visualisation of rabbit IgG with Alexa Fluor 594 secondary antibodies (red), (c) bright light (d) overlay of pictures a, b, and c. Bar: 5 µm. (TIF) [file pone.0079920.s001.tif]

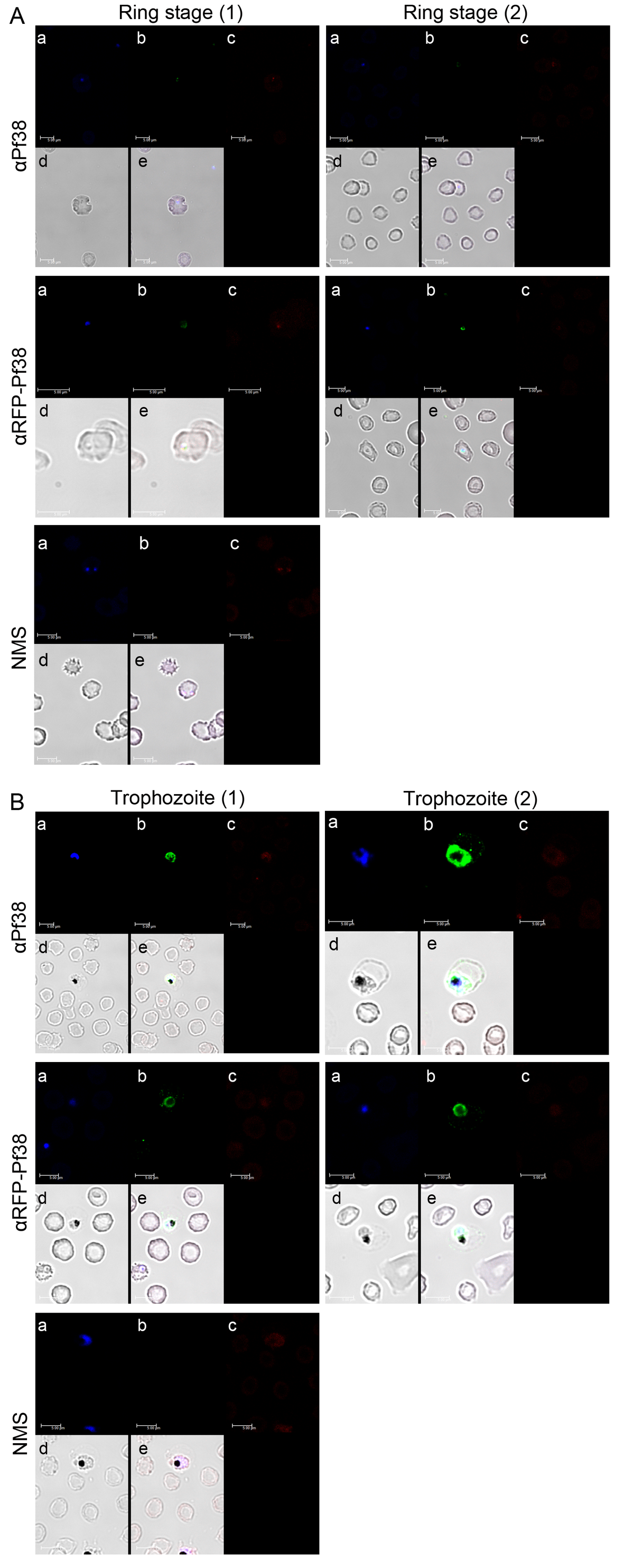

Supplement: Figure S2 — Immunofluorescence assay of NF54 parasites in ring stage and trophozoite stage. For IFAs, P. falciparum NF54 parasites in ring stage (A) and trophozoite stage (B) were fixed with methanol on the surface of a slide. αPf38: Detection was performed using the protein G-purified αPf38 murine IgG fraction. αRFP-Pf38: Detection was performed using the protein G-purified αRFP-Pf38 murine IgG fraction. NMS: Detection was performed using the protein G-purified murine IgG fraction of neutral mouse serum. As a positive control, a rabbit αAMA-1 IgG fraction was used. (a) Nuclei were stained with Hoechst 33342. (b) Visualisation of murine IgG with Alexa Fluor 488 secondary antibodies (green), (c) visualisation of rabbit IgG with Alexa Fluor 594 secondary antibodies (red), (d) bright light (e) overlay of pictures a, b, c and d. Bar: 5 µm. (TIF) [file pone.0079920.s002.tif]

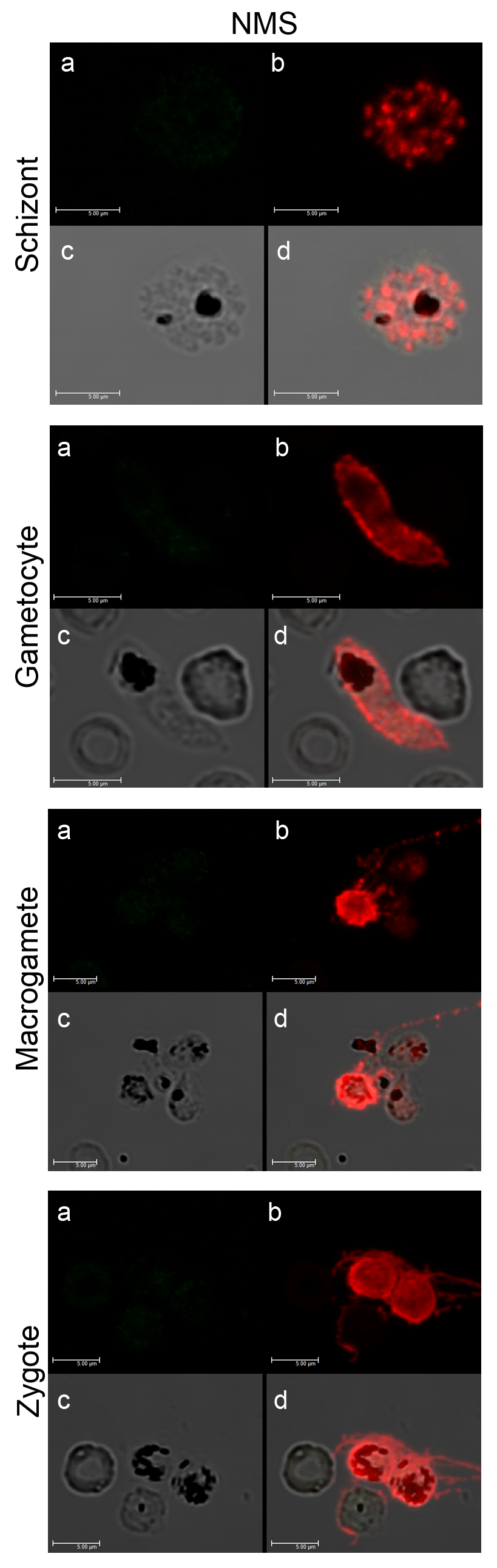

Supplement: Figure S3 — Negative controls for Immunofluorescence assays depicted in Figure 4 and Figure S1. For IFAs, P. falciparum NF54 parasites in the schizont, gametocyte, macrogamete and zygote stages were fixed with methanol on the surface of a slide. Detection was performed using the protein G-purified murine IgG fraction of neutral mouse serum. As a positive control, a rabbit αAMA-1 IgG fraction was used for schizonts, and rabbit αPfs25 serum was used for the sexual stages. (a) Visualisation of murine IgG with Alexa Fluor 488 secondary antibodies (green), (b) visualisation of rabbit IgG with Alexa Fluor 594 secondary antibodies (red), (c) bright light (d) overlay of pictures a, b, and c. Bar: 5 µm. (TIF) [file pone.0079920.s003.tif]

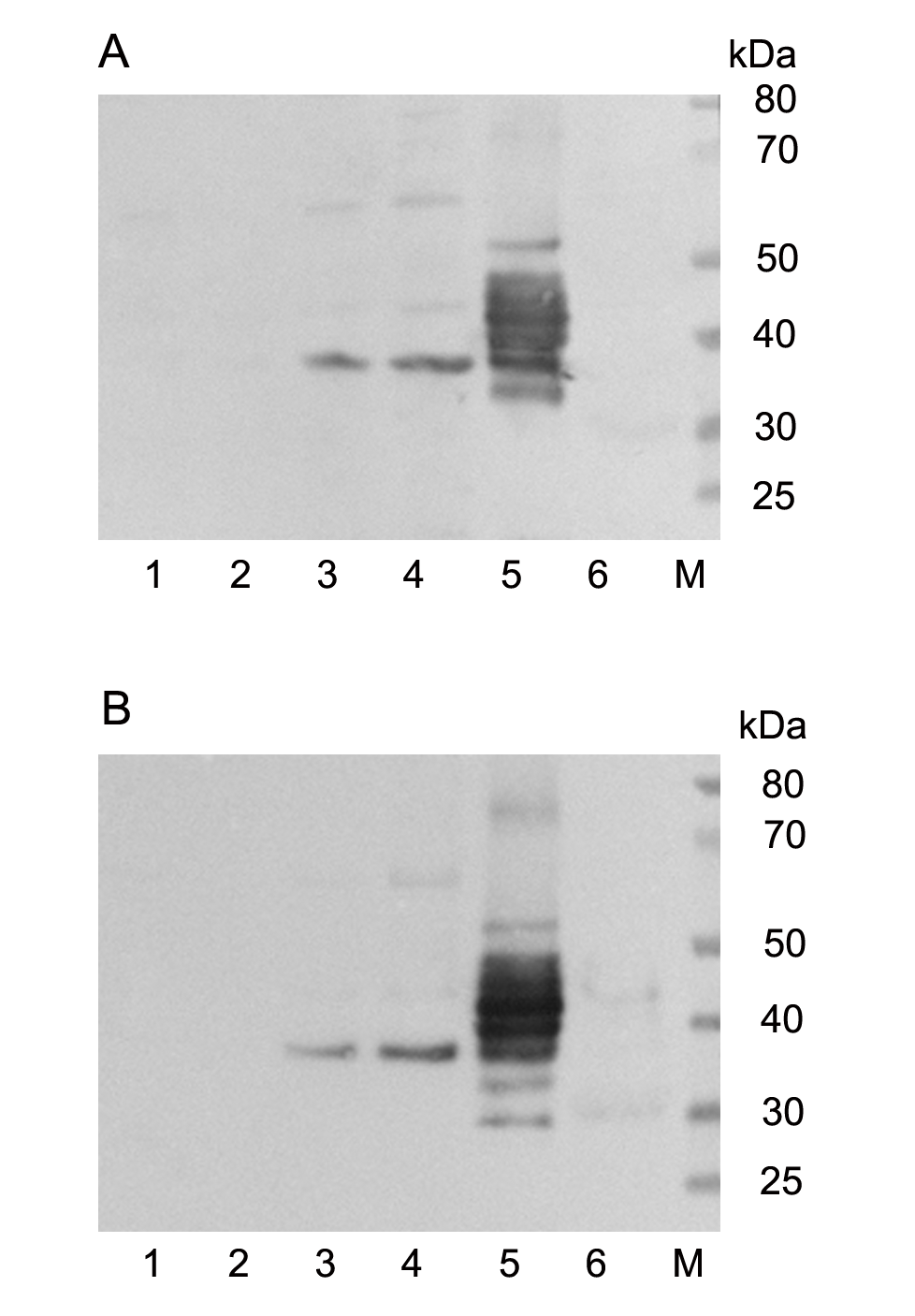

Supplement: Figure S4 — Immunoblot to prove specificity of generated αPf38 sera. Parasite preparations and plant produced Pf38 were run on SDS-PAGE gel and subsequently blotted. Detection of Pf38 was performed using murine αPf38 IgG (A) or murine αRFP-Pf38 IgG (B) and an alkaline phosphatase-labelled goat αmouse antiserum with nitroblue tetrazolium/5-bromo-4-chloro-3-indolyl-phosphate solution as the substrate. For all asexual stages 4.5×10^6^ parasites were used, while the gametocyte preparation contained 5×10^5^ gametocytes. 1: Asexual parasites 12 h after invasion 2: Asexual parasites 24 h after invasion 3: Asexual parasites 36 h after invasion 4: Asexual parasites 48 h after invasion 5: plant produced Pf38 (600 ng) 6: gametocyte preparation M: PageRuler™ Prestained protein ladder (Fermentas). (TIF) [file pone.0079920.s004.tif]
