# Supplementary material for: Plant-Based Production of Recombinant Plasmodium Surface Protein Pf38 and Evaluation of its Potential as a Vaccine Candidate
Source: PLoS One. 2013 Nov 21;8(11):e79920. doi: 10.1371/journal.pone.0079920 (PMC3836784; doi:10.1371/journal.pone.0079920)
Supplement: Text S1 — Preparation and results of immunoblot depicted in Figure S4. (DOCX) [file pone.0079920.s005.docx]

### Text S1:

Immunoblots for verification of specificity of αPf38 antibodies.

To prepare asexual parasites for immunoblot analysis 3D7A lysates from 4 time points of the blood stage reproduction cycle were prepared as described before with modifications [S1]. In short 3D7A parasites were synchronized at the ring stage using by 5% sorbitol solution for 10 min. This treatment was repeated during three subsequent reproduction cycles. In order to collect ring stage parasites, trophozoites and early schizonts, parasites from 60ml parasite culture (5% hematocrit, 2% parasitemia) were collected at 12h, 24h and 36 h after reinvasion. The red blood cells (RBCs) were pelleted by centrifugation at 500 x g for 5 min and subsequently lysed in a solution of 0.075% saponin in PBS for 10 min. Free parasites were washed with PBS and subsequently with ice-cold PBS until the solution was colorless. Parasites were taken up in 3 pellet volumes of 1x SDS-PAGE loading buffer.

For the late schizont stage, 40 hours post invasion, parasites were enriched by centrifugation in 70% Percoll as previously described [S2]. Subsequently, parasites were cultivated for 8 hours in 10µM E64 (Sigma) in 30ml standard malaria culture medium, a treatment which arrests the schizonts at the very latest stage before erythrocyte burst [S3]. Subsequently, remaining RBCs were removed using 0.075% saponin and resulting pure parasites were thoroughly washed using ice-cold PBS. Lysates were prepared as described above.

To prepare early gametocyte stages (stages 1 and 2), parasites were treated for 5 consecutive days with 50mM N-acetylglucosamine (GlcNac) [S4] with daily changes of medium. GlcNac is toxic to trophozoites and schizonts and loss of these stages after 5 days was observed by giemsa smears and light microscopy. Afterwards gametocytes were purified from most of the accompanying uninfected erythrocytes by Percoll density gradient centrifugation as described elsewhere [S5], suspended in PBS and stored at ‑20°C till further use.

For immunoblot analysis two 4-12% gradient NuPAGE gels (Lifetechnologies) were run containing the four preparations of asexual parasites (from different time points of the life cycle) at 4.5x10^6^ parasites, 600ng plant-produced and purified Pf38 and the preparation of gametocytes (5x 10^5^ parasites). Gel electrophoresis was performed under non-reducing conditions. Gels were blotted and blocked. Blot A (Pf38) and B (RFP-Pf38) were treated with the murine IgG preparations used for all other assays in this work at a dilution of 1:200 in PBS. For the secondary antibody, an alkaline phosphatase-labelled goat αmouse antiserum (Jackson ImmunoResearch) was used, and nitroblue tetrazolium/5-bromo-4-chloro-3-indolyl-phosphate solution was utilised as the substrate.

The resulting immunoblots A and B are shown in figure S4. They show that both the αPf38 antibodies and the αRFP-Pf38 antibodies detect Pf38 in asexual parasite preparations as well as the plant produced Pf38. The specificity of the generated antibodies is therefore confirmed.

References:

S1. [Mordmüller B](http://www.ncbi.nlm.nih.gov/pubmed?term=Mordm%C3%BCller%20B%5BAuthor%5D&cauthor=true&cauthor_uid=16616382), [Fendel R](http://www.ncbi.nlm.nih.gov/pubmed?term=Fendel%20R%5BAuthor%5D&cauthor=true&cauthor_uid=16616382), [Kreidenweiss A](http://www.ncbi.nlm.nih.gov/pubmed?term=Kreidenweiss%20A%5BAuthor%5D&cauthor=true&cauthor_uid=16616382), [Gille C](http://www.ncbi.nlm.nih.gov/pubmed?term=Gille%20C%5BAuthor%5D&cauthor=true&cauthor_uid=16616382), [Hurwitz R](http://www.ncbi.nlm.nih.gov/pubmed?term=Hurwitz%20R%5BAuthor%5D&cauthor=true&cauthor_uid=16616382), et al. (2006) Plasmodia express two threonine-peptidase complexes during asexual development**.** Mol Biochem Parasitol 14:79-85.

S2. [Radfar A](http://www.ncbi.nlm.nih.gov/pubmed?term=Radfar%20A%5BAuthor%5D&cauthor=true&cauthor_uid=20010926), [Méndez D](http://www.ncbi.nlm.nih.gov/pubmed?term=M%C3%A9ndez%20D%5BAuthor%5D&cauthor=true&cauthor_uid=20010926), [Moneriz C](http://www.ncbi.nlm.nih.gov/pubmed?term=Moneriz%20C%5BAuthor%5D&cauthor=true&cauthor_uid=20010926), [Linares M](http://www.ncbi.nlm.nih.gov/pubmed?term=Linares%20M%5BAuthor%5D&cauthor=true&cauthor_uid=20010926), [Marín-García P](http://www.ncbi.nlm.nih.gov/pubmed?term=Mar%C3%ADn-Garc%C3%ADa%20P%5BAuthor%5D&cauthor=true&cauthor_uid=20010926), et al. (2009) Synchronous culture of *Plasmodium falciparum* at high parasitemia levels. [Nat](http://www.ncbi.nlm.nih.gov/pubmed/?term=20010926) Protoc: 1899-1915.

S3. [Boyle MJ](http://www.ncbi.nlm.nih.gov/pubmed?term=Boyle%20MJ%5BAuthor%5D&cauthor=true&cauthor_uid=20660744), [Wilson DW](http://www.ncbi.nlm.nih.gov/pubmed?term=Wilson%20DW%5BAuthor%5D&cauthor=true&cauthor_uid=20660744), [Richards JS](http://www.ncbi.nlm.nih.gov/pubmed?term=Richards%20JS%5BAuthor%5D&cauthor=true&cauthor_uid=20660744), [Riglar DT](http://www.ncbi.nlm.nih.gov/pubmed?term=Riglar%20DT%5BAuthor%5D&cauthor=true&cauthor_uid=20660744), [Tetteh KK](http://www.ncbi.nlm.nih.gov/pubmed?term=Tetteh%20KK%5BAuthor%5D&cauthor=true&cauthor_uid=20660744), [et](http://www.ncbi.nlm.nih.gov/pubmed?term=Beeson%20JG%5BAuthor%5D&cauthor=true&cauthor_uid=20660744) al. (2010) Isolation of viable Plasmodium falciparum merozoites to define erythrocyte invasion events and advance vaccine and drug development. [Proc Natl Acad Sci U S A](http://www.ncbi.nlm.nih.gov/pubmed/?term=20660744).107:14378-14383.

S4. Fivelman QL, McRobert L, Sharp S, Taylor CJ, Saeed M, et al.(2007) Improved synchronous production of *Plasmodium falciparum* gametocytes in vitro. Mol Biochem Parasitol 154: 119-123.

S5. Kariuki MM, Kiaira JK, Mulaa FK, Mwangi JK, Wasunna MK, et al. (1998) *Plasmodium* *falciparum*: Purification of the various gametocyte developmental stages from in vitro cultivated parasite. Am J Trop Med Hyg 59: 505-508.
